# Understanding SARS‐CoV‐2 endocytosis for COVID‐19 drug repurposing

**DOI:** 10.1111/febs.15369

**Published:** 2020-06-02

**Authors:** Oleg O. Glebov

**Affiliations:** ^1^ Institute of Neuroregeneration and Neurorehabilitation Qingdao University Qingdao Shandong China; ^2^ Department of Old Age Psychiatry The Institute of Psychiatry, Psychology and Neuroscience King’s College London London England UK

**Keywords:** COVID‐19, drug repurposing, endocytosis, membrane trafficking, SARS‐CoV‐2

## Abstract

The quest for the effective treatment against coronavirus disease 2019 pneumonia caused by the severe acute respiratory syndrome (SARS)‐coronavirus 2(CoV‐2) coronavirus is hampered by the lack of knowledge concerning the basic cell biology of the infection. Given that most viruses use endocytosis to enter the host cell, mechanistic investigation of SARS‐CoV‐2 infection needs to consider the diversity of endocytic pathways available for SARS‐CoV‐2 entry in the human lung epithelium. Taking advantage of the well‐established methodology of membrane trafficking studies, this research direction allows for the rapid characterisation of the key cell biological mechanism(s) responsible for SARS‐CoV‐2 infection. Furthermore, 11 clinically approved generic drugs are identified as potential candidates for repurposing as blockers of several potential routes for SARS‐CoV‐2 endocytosis. More broadly, the paradigm of targeting a fundamental aspect of human cell biology to protect against infection may be advantageous in the context of future pandemic outbreaks.

## Graphical abstract

A key step in COVID‐19 pathogenesis is the entry of the SARS‐CoV‐2 virus into the airway epithelial cells; this process employs the cellular mechanisms of endocytosis, and therefore targeting these mechanisms has therapeutic potential for treating COVID‐19. Generic drugs that have been shown to inhibit endocytosis represent promising candidates for development of affordable anti‐COVID‐19 treatments.

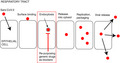

**Abbreviations**
COVID‐19: coronavirus disease 2019
SARS: severe acute respiratory syndrome
CoV‐2: coronavirus 2
CoV: coronavirus
MERS: Middle East respiratory syndrome
ACE2: angiotensin‐converting enzyme 2
CtBP: C‐terminal binding protein
AP2: adaptor protein 2
AP180: adaptor protein 180
Arf: adenosine diphosphate‐ribosylation factor
Rac1: Ras‐related C3 botulinum toxin substrate 1
Cdc42: cell division control protein 42

## Introduction

At the time of writing this piece, the pandemic of the atypical pneumonia coronavirus disease 2019 (COVID‐19) caused by a novel coronavirus severe acute respiratory syndrome (SARS)‐coronavirus 2 (CoV‐2) has spread globally, with more than 4 million cases and close to 300 000 deaths (https://www.worldometers.info/coronavirus/). This current epidemiological, economical and potentially political disaster in the making follows in the wake of earlier outbreaks of respiratory infections SARS and Middle East respiratory syndrome (MERS), also caused by coronaviruses [1]. There is currently no known cure for COVID‐19, and available medical treatment is largely limited to alleviation of the respiratory dysfunction associated with the severe course of the disease.

As a result of this, an enormous effort across biomedical sciences is mounting to develop therapeutic means of treating COVID‐19. One key area of this development is vaccination. As of May 11, there were five candidate vaccines in clinical evaluation, with as many as 71 in preclinical stage [2]. Yet necessities of the research and development process dictate that none of the above – in the best possible scenario – will be clinically available earlier than in 18 months’ time [3]. Given the unprecedented scale of the current outbreak, there is immense pressure to find faster and cheaper therapeutic solutions to this rapidly worsening global health crisis.

There is a broad agreement that perhaps the best way to produce an affordable treatment on a faster timescale is to repurpose existing drugs, and much interest is directed this way, especially focusing on drugs with previously demonstrated antiviral capabilities [4]. However, lack of the basic understanding of the cell biology of SARS‐CoV‐2 infection limits the efficacy of such an approach. Although recent years have yielded some insight into the cell biology of the related human virus SARS‐CoV, and the two viruses may bind to the same surface receptor protein angiotensin‐converting enzyme 2 (ACE2) [5, 6, 7, 8], their genomes still differ by 20% [8], and the associated epidemiological picture is very different indeed. Furthermore, the extent of ACE2 protein expression in the airways is subject to some controversy [9, 10, 11, 12, 13], while preliminary evidence hints that other surface receptors may be involved in SARS‐CoV‐2 infection [14]. The resulting gap in knowledge forms a major obstacle on the road towards effective drug repurposing for treating COVID‐19.

## The many unknowns of SARS‐CoV‐2 cell entry

**Fig. 1:**
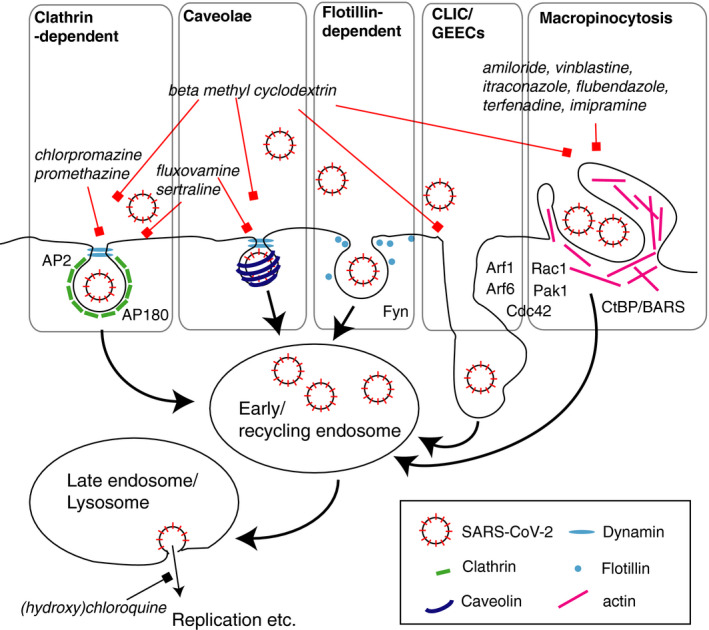
A simplified representation of endocytic pathways potentially involved in internalisation of SARS‐CoV‐2. Selected endocytic pathways are enclosed in grey boxes. Selected key structural and regulatory proteins are shown graphically or mentioned by name. Thick arrows represent the directional flow of membrane trafficking pathways. A thin black arrow represents viral trajectory following the completion of its putative endocytic itinerary. Red lines with squares at their ends indicate putative pharmacological blockade of processes. Black line with a square at its end indicates the suggested effect of chloroquine and hydroxychloroquine on viral fusion in the endosome. The names of the candidate drug blockers are italicised.

The critical step in any viral infection involves penetration of the viral particles into the cytosol; to this end, most viruses take advantage of the endocytic membrane trafficking of the host cell [15]. While the canonical endocytic pathways in mammalian cells involve budding of clathrin‐coated vesicles through the action of a large GTPase dynamin, there are also multiple pathways of noncanonical endocytosis such as caveolae, flotillin‐1‐associated endocytosis, Clathrin‐independent carrier/glycosylphosphatidylinositol‐anchored protein‐enriched endosomal compartment endocytosis and macropinocytosis [16], and viruses have been shown to use all of these [15] (Fig. 1). Mechanistic details of these pathways may vary considerably between cell types, and diversity of endocytosis in airway epithelium is currently poorly understood. Understanding endocytic viral entry in the respiratory tract may therefore offer a promising therapeutic strategy to treat viral infections.

As far as coronaviruses are concerned, current evidence suggests that the mode of entry can vary between viruses and host cell types, and can include clathrin‐dependent endocytosis [17], caveolae [18], and clathrin‐ and caveolae‐independent mechanism involving lipid rafts [19, 20]. Fusion at the cell surface, while proposed as a potential entry mechanism [5], was not directly demonstrated. Both SARS‐CoV and SARS‐CoV‐2 appear to require endocytosis in cultured cell lines [6, 19, 20, 21], and SARS‐CoV may be endocytosed via more than one pathway depending on the cell line [19, 21]. However, there is little knowledge regarding the details of the epidemiologically relevant mode of viral entry, that is in the human lung. It is therefore imperative to consider SARS‐CoV‐2 endocytic trafficking in context of the correct host cell type.

Preliminary evidence suggests that infection with SARS‐CoV‐2 may begin in the upper respiratory tract, for example in the nasal epithelium, which expresses the highest levels of SARS‐CoV‐2 receptors [10]. The most significant morbidity and indeed mortality, however, appear to be associated with a further, severe stage of the disease, when infection spreads to the lower airways of the lung, resulting in respiratory failure and possibly multiorgan failure due to cytokine storm [22]. Although the histopathology data are still scant, two case studies and preliminary findings from a primate disease model indicate that the lower airway SARS‐CoV‐2 infection may specifically affect the alveolar epithelial cells termed pneumocytes [23, 24, 25]. Clearly, blockade of the SARS‐CoV‐2 entry at either of these stages is likely to yield considerable therapeutic benefits.

## Endocytic pathways in respiratory epithelial cells

To establish which endocytic pathways are likely to operate in nasal epithelial cells and pneumocytes, some clues can be gleaned from the local expression profile for the key endocytic proteins as evidenced by the Human Protein Atlas (https://www.proteinatlas.org/). Nasal epithelial cells abundantly express a wide variety of endocytic markers, suggesting the presence of multiple active pathways, in agreement with ultrastructural evidence [26]. Conversely, pneumocytes show a more restricted expression pattern, with some key proteins associated with conventional endocytosis either low or missing. Importantly, a large GTPase, dynamin, which is required for endocytosis through clathrin‐coated vesicles and caveolae, is abundant in nasal epithelium but undetectable in pneumocytes. In contrast, there are medium‐to‐high expression levels of proteins involved in macropinocytosis, such as C‐terminal binding protein (CtBP)1 & 2 and P21‐activated kinase 1. Therefore, macropinocytosis may be an important endocytic pathway in pneumocytes.

The potentially distinct endocytic profile of upper and lower airways is reflected in the relevant infection mechanisms for known pathogens. Upper respiratory epithelium can be infected by numerous airborne pathogens through multiple pathways of dynamin‐dependent and dynamin‐independent endocytosis [15]. Conversely, pneumocyte infection is much less well understood; however, entry through macropinocytosis has been consistently demonstrated for airborne bacterial and viral pathogens such as *Mycobacterium tuberculosis* [27], *Francisella tularensis* [28], influenza A virus [29], adenovirus adenovirus serotype 35 [30] and *Haemophilus influenzae* [31], as well as for various nanoparticles [32, 33]. Intriguingly, macropinocytosis in an alveolar epithelial cell line is upregulated by water‐pipe smoke condensate, suggesting a possible mechanism underlying association of COVID‐19 morbidity with smoking [34].

Taken together, the above evidence suggests that SARS‐CoV‐2 may employ distinct endocytic pathways for cell entry in the upper and lower respiratory tract (Fig. 1). What are these pathways? To address this question directly, we need to investigate the cell biology of SARS‐CoV‐2 endocytosis in detail.

## Potential experimental model for investigating the key events of SARS‐CoV‐2 cell entry *in vitro*

A suitable starting model for initial investigation of SARS‐CoV‐2 endocytosis can involve established immortalised cell lines derived from the respiratory tract epithelium (https://www.atcc.org/~/media/PDFs/Cancer%20and%20Normal%20cell%20lines%20tables/Lung%20cancer%20and%20normal%20cell%20lines.ashx). These lines provide several key methodological advantages: they are well‐characterised, easy to maintain using standard cell culture protocols and retain the key characteristics of the primary cell type of origin. For emulation of the respiratory tract environment, the cell lines can be grown in an air–liquid interface culture as described before [35, 36].

Immortalised cell cultures offer a simple and cost‐effective platform for investigation of cell biology. There are, however, important caveats associated with immortalised cell cultures *in vitro*, which need addressing and further validation. One key consideration is expression profile, in which a cell line may be different from that in the original tissue. This can be especially relevant with regard to membrane trafficking, where discrepancy in expression of certain key proteins may affect the organisation of the whole network: for example, the lung cell line A549 can express multiple isoforms of dynamin [37], which is not the case in pneumocytes in human lung tissue.

As a result of this, any findings arising from the cell lines will need to be investigated further in a more expensive, but clinically relevant system of primary cells. Cell preparations for both lower and upper respiratory tract are commercially available [38, 39]. Preliminary evidence shows that such systems can be efficiently infected with SARS‐CoV‐2 [40]. Alternatively, cells can be directly obtained from human subjects, for example nasal epithelium, or alveolar epithelial cells can be isolated from surgically resected lung tissue material [41]. Regardless of that, validation of findings in primary cells will be a key step in investigation.

## Experimental interrogation of SARS‐CoV‐2 membrane trafficking

Investigation of membrane trafficking of SARS‐CoV‐2 requires a probe that can adequately recapitulate the intracellular itinerary of the virus. Using active, clinically isolated live virus samples would of course allow a closest approximation. However, a major drawback of this approach is a highly infectious nature of the virus, necessitating the use of a Biosafety Level 3 Laboratory. An alternative approach would involve pseudoviruses, combining viral surface proteins responsible for cell receptor binding. The lack of SARS‐CoV‐2 genetic material renders them incapable of replication, allowing work in a Biosafety Level 2 Laboratory. Pseudoviruses have been successfully used before to investigate trafficking of SARS‐CoV and MERS‐CoV [5, 19], and SARS‐CoV‐2 pseudovirus models have been recently published [6, 7].

For infection, the viral probe will be added to the cells for different lengths of time. In order to determine the endocytic pathway(s) involved in SARS‐CoV‐2 endocytosis, one can employ standard methods of multicolour fluorescence immunocytochemistry, light microscopy and colocalisation analysis. The proportion of the internalised virus colocalising with the classical markers of membrane trafficking compartments will indicate the intracellular itinerary of the virus [42]. For this approach, multiple well‐characterised antibody markers for intracellular compartments, for example early endosomes, late endosomes and lysosomes are available.

For a more detailed investigation of the endocytic route of the virus, viral infection can be combined with uptake of typical cargoes for different endocytic pathways. This approach would allow tracking of the virus in relation to other endocytic pathways and also to investigate the effect of viral infection on the general membrane trafficking network of the host cell. Suitable cargo molecules include transferrin for clathrin‐dependent endocytosis [43], cholera toxin B subunit for caveolae [44], and dextran for fluid‐phase macropinocytosis and flotillin‐dependent endocytosis [45].

What is the relative contribution of different endocytic pathways to SARS‐CoV‐2 infection? To address this question, loss‐of‐function analysis can be performed, involving knockdown of the key regulatory proteins for different endocytic pathways, for example dynamin 2, caveolin‐1, flotillin‐1 and CtBP [16, 44] (Fig. 1). The resulting findings can be further corroborated using available pharmacological blockers of endocytic pathways, including dynasore and pitstop for clathrin‐dependent endocytosis, nystatin for caveolae, methyl‐beta‐cyclodextrin for dynamin‐independent endocytosis and amiloride for macropinocytosis. However, care must be exercised in interpretation of these results due to the off‐target effects of these agents, in which endocytic pathways other than the target pathway may be inhibited [46, 47, 48]. Taken together, the combination of adequate cell models with the newly developed SARS‐CoV‐2 toolkit and established tools of membrane trafficking research is well‐poised to deliver a key insight into the mechanisms underlying COVID‐19 infection.

## Repurposing previously approved drugs for blocking SARS‐CoV‐2 endocytosis

Two broad approaches to developing drugs against SARS‐CoV‐2 involve targeting the biology of the host versus targeting the biology of the virus. One clear drawback associated with global perturbation of a general cell biological function is the potential for unwelcome side effects, whether in the same target tissue or elsewhere in the body. Conversely, a key advantage of focusing on the cell biological mechanism is the relatively immutability of the host cell compared to virus, which may evolve drug resistance due to its high mutation rate [49, 50]. Furthermore, considering that various viruses may use the same endocytic pathways of the host cell [15], targeting viral entry at the point of endocytosis holds a more general promise for the development of broad‐spectrum antiviral drugs [51].

Once the endocytic route of SARS‐CoV‐2 in primary lung epithelial cells has been established, this insight can be capitalised upon for translational needs. Specifically, a cell‐based model of SARS‐CoV‐2 infection [40] will lend itself to establishment of a screening platform for drug development, aiming to identify compounds capable of blocking or hindering SARS‐CoV‐2 endocytic entry. There is already vivid interest in chloroquine and hydroxychloroquine, two related drugs that may block viral entry through endosomal acidification (Fig. 1), although evidence for their effectiveness is currently scant and safety hazards are a major concern [4, 52, 53]. Of note, compounds showing even partial blockade of SARS‐CoV‐2 endocytosis may be of value, as clinical evidence suggests that viral load may correlate with the severity of the disease progression.

Large‐scale screening of this sort will require considerable time and effort; in the meantime, a short‐term screening of a limited number of candidate drugs is highly warranted. To this end, the range of candidates can be limited to the following 11 approved drugs (Table 1, Fig. 1). Although most of these drugs are not currently prescribed against infection, they have shown ability to block various pathways of endocytosis in cell culture and may therefore have potential for blocking SARS‐CoV‐2 endocytosis. Another addition to this list is a cholesterol chelating agent methyl‐beta‐cyclodextrin, used as a carrier to enhance bioavailability in drug formulations and generally recognised as safe by the Food and Drug Administration (USA).

**Table 1:** Previously approved drugs to be tested for blockade of SARS‐CoV‐2 endocytosis in respiratory epithelium.

Name	Primary indication	Potential direct delivery into the respiratory tract	Endocytic pathway affected
Chlorpromazine	Antipsychotic		Clathrin, other [48]
Fluvoxamine	Antidepressant		Dynamin [55]
Sertraline	Antidepressant		Dynamin [56]
Promethazine	Antihistamine	Aerosol [57]	Clathrin [58]
Nystatin	Antifungal	Aerosol [59]	Caveolae [48]
Amiloride	Diuretic	Inhalation [60]	Macropinocytosis
Vinblastine	Microtubule formation inhibitor		Macropinocytosis [61], Clathrin [62]
Itraconazole	Antifungal	Inhaled powder (Phase 2) [63, 64]	Macropinocytosis [61]
Flubendazole	Antihelminthic		Macropinocytosis [61]
Terfenadine	Histamine H1 receptor antagonist		Macropinocytosis [61]
Imipramine	Antidepressant		Macropinocytosis [61]
Beta‐methyl cyclodextrin	Bioavailability enhancer	Intranasal [65]	Multiple

Importantly, 5 out of 11 drugs (chlorpromazine, nystatin, amiloride, vinblastine and itraconazole) are on the World Health Organization’s List of Essential Medicines, ensuring their wide availability across the world. Also, several of the above have been tested (or are being tested) for topical delivery into the respiratory tract (Table 1). These factors will be favourable for the rapid integration of the successful drugs into global clinical practice, subject to development of relevant clinical and epidemiological applications.

## Conclusion

Despite frenzied efforts to find effective treatments for COVID‐19, very little is currently known about the fundamental cell biology of the SARS‐CoV‐2 infection. The pivotal event underpinning SARS‐CoV‐2 infection is likely endocytosis of viral particles; therefore, blockade of this process may provide for a major therapeutic breakthrough. The tools of membrane trafficking research can be readily applied to rapidly characterise the endocytic route of virus entry in a cell‐based model of disease, providing a key insight into the disease mechanism (Fig. 1). Crucially, the same model can be used as a screening platform for rapid repurposing of existing approved drugs as blockers of SARS‐CoV‐2 endocytic entry (Table 1). In the longer term, insight into the poorly understood membrane trafficking mechanisms of the airway epithelium will aid development of novel drugs.

The generalised strategy of targeting the membrane trafficking of the host cell biology to prevent viral infection holds great promise for future therapeutic applications, considering the existence of other zoonotic coronaviruses capable of binding to human cells [54]. More broadly, rapid repurposing of approved drugs in cell‐based screening platforms is poised to play an instrumental role in future‐proofing global health care against the interspecies transmission events. In the age of unparalleled spending on pharmaceutical research and development, humankind cannot afford to neglect one of its most valuable allies in the fight against the current and the future pandemics – the basic foundations of human cell biology.

## Conflict of interest

The authors declare no conflict of interest.

## References

[1] Park M , Thwaites RS & Openshaw PJM (2020) COVID‐19: lessons from SARS and MERS. Eur J Immunol 50, 308–311.

[2] WHO (2020) Coronavirus disease (COVID‐2019) R&D. WHO.

[3] Coronavirus vaccine: when will it be ready? | World news | The Guardian. Available at: https://www.theguardian.com/world/2020/apr/01/coronavirus‐vaccine‐when‐will‐it‐be‐ready‐covid‐19. (Accessed 1st April 2020).

[4] Dong L , Hu S & Gao J (2020) Discovering drugs to treat coronavirus disease 2019 (COVID‐19). Drug Discov Ther 14, 58–60. 32147628 10.5582/ddt.2020.01012

[5] Lu L , Liu Q , Zhu Y , Chan KH , Qin L , Li Y , Wang Q , Chan JF , Du L , Yu F *et al*. (2014) Structure‐based discovery of Middle East respiratory syndrome coronavirus fusion inhibitor. Nat Commun 5, 3067. 24473083 10.1038/ncomms4067 PMC7091805

[6] Ou X , Liu Y , Lei X , Li P , Mi D , Ren L , Guo L , Guo R , Chen T , Hu J *et al*. (2020) Characterization of spike glycoprotein of SARS‐CoV‐2 on virus entry and its immune cross‐reactivity with SARS‐CoV. Nat Commun 11, 1620. 32221306 10.1038/s41467-020-15562-9 PMC7100515

[7] Hoffmann M , Kleine‐Weber H , Schroeder S , Krüger N , Herrler T , Erichsen S , Schiergens TS , Herrler G , Wu N‐H , Nitsche A *et al*. (2020) SARS‐CoV‐2 cell entry depends on ACE2 and TMPRSS2 and is blocked by a clinically proven protease inhibitor. Cell 181, 271–280. 32142651 10.1016/j.cell.2020.02.052 PMC7102627

[8] Zhou P , Yang X‐L , Wang X‐G , Hu B , Zhang L , Zhang W , Si H‐R , Zhu Y , Li B , Huang C‐L *et al*. (2020) A pneumonia outbreak associated with a new coronavirus of probable bat origin. Nature 579, 270–273. 32015507 10.1038/s41586-020-2012-7 PMC7095418

[9] Hikmet F , Mear L , Uhlen M & Lindskog C (2020) The protein expression profile of ACE2 in human tissues . bioRxiv, 10.1101/2020.03.31.016048 PMC7383091 32715618

[10] Sungnak W , Huang N , Bécavin C , Berg M & Network HLB (2020) SARS‐CoV‐2 entry genes are most highly expressed in nasal goblet and ciliated cells within human airways.

[11] Ziegler CGK , Allon SJ , Nyquist SK , Mbano IM , Miao VN , Tzouanas CN , Cao Y , Yousif AS , Bals J , Hauser BM *et al*. (2020) SARS‐CoV‐2 receptor ACE2 is an interferon‐stimulated gene in human airway epithelial cells and is detected in specific cell subsets across tissues. Cell, 181, 1016–1035. 32413319 10.1016/j.cell.2020.04.035 PMC7252096

[12] Pinto BG , Oliveira AE , Singh Y , Jimenez L , Goncalves AN , Ogava RL , Creighton R , Peron JP & Nakaya HI (2020) ACE2 expression is increased in the lungs of patients with comorbidities associated with severe COVID‐19. medRxiv. 10.1101/2020.03.21.20040261. PMC7377288 32526012

[13] Li G , He X , Zhang L , Ran Q , Wang J , Xiong A , Wu D , Chen F , Sun J & Chang C (2020) Assessing ACE2 expression patterns in lung tissues in the pathogenesis of COVID‐19. J Autoimmun 102463, 10.1016/j.jaut.2020.102463. 32303424 PMC7152872

[14] Wang K , Chen W , Zhou YS , Lian JQ , Zhang Z , Du P , Gong L , Zhang Y , Cui HY , Geng JJ *et al*. (2020) SARS‐CoV‐2 invades host cells via a novel route: CD147‐spike protein. bioRxiv. 10.1101/2020.03.14.988345 PMC7714896 33277466

[15] Mercer J , Schelhaas M & Helenius A (2010) Virus entry by endocytosis. Annu Rev Biochem 79, 803–833. 20196649 10.1146/annurev-biochem-060208-104626

[16] Doherty GJ & McMahon HT (2009) Mechanisms of endocytosis. Annu Rev Biochem 78, 857–902. 19317650 10.1146/annurev.biochem.78.081307.110540

[17] Milewska A , Nowak P , Owczarek K , Szczepanski A , Zarebski M , Hoang A , Berniak K , Wojarski J , Zeglen S , Baster Z *et al*. (2018) Entry of human coronavirus NL63 into the cell. J Virol 92. 10.1128/JVI.01933-17 PMC5774871 29142129

[18] Szczepanski A , Owczarek K , Milewska A , Baster Z , Rajfur Z , Mitchell JA & Pyrc K (2018) Canine respiratory coronavirus employs caveolin‐1‐mediated pathway for internalization to HRT‐18G cells. Vet Res 49, 55. 29970183 10.1186/s13567-018-0551-9 PMC6029178

[19] Wang H , Yang P , Liu K , Guo F , Zhang Y , Zhang G & Jiang C (2008) SARS coronavirus entry into host cells through a novel clathrin‐ and caveolae‐independent endocytic pathway. Cell Res 18, 290–301. 18227861 10.1038/cr.2008.15 PMC7091891

[20] Lu Y , Liu DX & Tam JP (2008) Lipid rafts are involved in SARS‐CoV entry into vero E6 cells. Biochem Biophys Res Commun 369, 344–349. 18279660 10.1016/j.bbrc.2008.02.023 PMC7092920

[21] Inoue Y , Tanaka N , Tanaka Y , Inoue S , Morita K , Zhuang M , Hattori T & Sugamura K (2007) Clathrin‐dependent entry of severe acute respiratory syndrome coronavirus into target cells expressing ACE2 with the cytoplasmic tail deleted. J Virol 81, 8722–8729. 17522231 10.1128/JVI.00253-07 PMC1951348

[22] Mehta P , McAuley DF , Brown M , Sanchez E , Tattersall RS & Manson JJ (2020) COVID‐19: consider cytokine storm syndromes and immunosuppression. Lancet 395, 1033–1034. 32192578 10.1016/S0140-6736(20)30628-0 PMC7270045

[23] Tian S , Hu W , Niu L , Liu H , Xu H & Xiao S‐Y (2020) Pulmonary pathology of early‐phase 2019 novel coronavirus (COVID‐19) pneumonia in two patients with lung cancer. J Thorac Oncol 15, 700–704. 32114094 10.1016/j.jtho.2020.02.010 PMC7128866

[24] Zhang H , Zhou P , Wei Y , Yue H , Wang Y , Hu M , Zhang S , Cao T , Yang C , Li M *et al*. (2020) Histopathologic changes and SARS–CoV‐2 immunostaining in the lung of a patient with COVID‐19. Ann Intern Med 172, 629–632. 32163542 10.7326/M20-0533 PMC7081173

[25] Rockx B , Kuiken T , Herfst S , Bestebroer T , Lamers MM , Munnink BB , de Meulder D , van Amerongen G , van den Brand J , Okba NM *et al*. (2020) Comparative pathogenesis of COVID‐19, MERS and SARS in a non‐human primate model . bioRxiv. 10.1101/2020.03.17.995639 PMC7164679 32303590

[26] Bannister LH & Dodson HC (1992) Endocytic pathways in the olfactory and vomeronasal epithelia of the mouse: ultrastructure and uptake of tracers. Microsc Res Tech 23, 128–141. 1421552 10.1002/jemt.1070230204

[27] García‐Pérez BE , Mondragón‐Flores R & Luna‐Herrera J (2003) Internalization of *Mycobacterium tuberculosis* by macropinocytosis in non‐phagocytic cells. Microb Pathog 35, 49–55. 12901843 10.1016/s0882-4010(03)00089-5

[28] Bradburne CE , Verhoeven AB , Manyam GC , Chaudhry SA , Chang EL , Thach DC , Bailey CL & van Hoek ML (2013) Temporal transcriptional response during infection of type II Alveolar Epithelial Cells with *Francisella tularensis* Live Vaccine Strain (LVS) supports a general host suppression and bacterial uptake by macropinocytosis. J Biol Chem 288, 10780–10791. 23322778 10.1074/jbc.M112.362178 PMC3624459

[29] de Vries E , Tscherne DM , Wienholts MJ , Cobos‐Jimenez V , Scholte F , Garcia‐Sastre A , Rottier PJ & de Haan CA (2011) Dissection of the influenza A virus endocytic routes reveals macropinocytosis as an alternative entry pathway. PLoS Pathog 7, e1001329. 21483486 10.1371/journal.ppat.1001329 PMC3068995

[30] Kalin S , Amstutz B , Gastaldelli M , Wolfrum N , Boucke K , Havenga M , DiGennaro F , Liska N , Hemmi S & Greber UF (2010) Macropinocytotic uptake and infection of human epithelial cells with species B2 adenovirus type 35. J Virol 84, 5336–5350. 20237079 10.1128/JVI.02494-09 PMC2863792

[31] López‐Gómez A , Cano V , Moranta D , Morey P , García del Portillo F , Bengoechea JA & Garmendia J (2012) Host cell kinases, α5 and β1 integrins, and Rac1 signalling on the microtubule cytoskeleton are important for non‐typable *Haemophilus influenzae* invasion of respiratory epithelial cells. Microbiology 158, 2384–2398. 22723286 10.1099/mic.0.059972-0

[32] dos Santos T , Varela J , Lynch I , Salvati A & Dawson KA (2011) Effects of transport inhibitors on the cellular uptake of carboxylated polystyrene nanoparticles in different cell lines. PLoS One 6, e24438. 21949717 10.1371/journal.pone.0024438 PMC3176276

[33] Sipos A , Kim K‐J , Sioutas C & Crandall ED (2019) Evidence for nanoparticle‐induced lysosomal dysfunction in lung adenocarcinoma (A549) cells. Int J Mol Sci 20, 5253. 10.3390/ijms20215253 PMC6861930 31652767

[34] Vardavas C & Nikitara K (2020) COVID‐19 and smoking: a systematic review of the evidence. Tob Induc Dis 18, 20. 32206052 10.18332/tid/119324 PMC7083240

[35] Wu J , Wang Y , Liu G , Jia Y , Yang J , Shi J , Dong J , Wei J & Liu X (2017) Characterization of air‐liquid interface culture of A549 alveolar epithelial cells. Brazilian J Med Biol Res 51, e6950. 10.1590/1414-431X20176950 PMC5731333 29267508

[36] Kreft ME , Jerman UD , Lasič E , Rižner TL , Hevir‐Kene N , Peternel L & Kristan K (2015) The characterization of the human nasal epithelial cell line RPMI 2650 under different culture conditions and their optimization for an appropriate *in vitro* nasal model. Pharm Res 32, 665–679. 25145337 10.1007/s11095-014-1494-0

[37] Reis CR , Chen P‐H , Bendris N & Schmid SL (2017) TRAIL‐death receptor endocytosis and apoptosis are selectively regulated by dynamin‐1 activation. Proc Natl Acad Sci USA 114, 504–509. 28049841 10.1073/pnas.1615072114 PMC5255607

[38] Huang S , Wiszniewski L , Constant S & Roggen E (2013) Potential of *in vitro* reconstituted 3D human airway epithelia (MucilAir™) to assess respiratory sensitizers. Toxicol Vitr 27, 1151–1156. 10.1016/j.tiv.2012.10.010 23089132

[39] Akimoto M , Hayashi J‐I , Nakae S , Saito H & Takenaga K (2016) Interleukin‐33 enhances programmed oncosis of ST2L‐positive low‐metastatic cells in the tumour microenvironment of lung cancer. Cell Death Dis 7, e2057. 26775708 10.1038/cddis.2015.418 PMC4816191

[40] Pizzorno A , Padey B , Julien T , Trouillet‐Assant S , Traversier A , Errazuriz‐Cerda E , Fouret J , Dubois J , Gaymard A , Lescure X *et al*. (2020) Characterization and treatment of SARS‐CoV‐2 in nasal and bronchial human airway epithelia. bioRxiv. 10.1101/2020.03.31.017889 PMC7373044 32835306

[41] Ehrhardt C , Kim K‐J & Lehr C‐M (2005) Isolation and culture of human alveolar epithelial cells. In Methods in Molecular Medicine ( Picot J , ed), vol. 107, pp. 207–216. Humana Press, Totowa, NJ. 15492374 10.1385/1-59259-861-7:207

[42] Kumari S , Mg S & Mayor S (2010) Endocytosis unplugged: multiple ways to enter the cell. Cell Res 20, 256–275. 20125123 10.1038/cr.2010.19 PMC7091825

[43] Glebov OO , Tigaret CM , Mellor JR & Henley JM (2015) Clathrin‐independent trafficking of AMPA receptors. J Neurosci 35, 4830–4836. 25810514 10.1523/JNEUROSCI.3571-14.2015 PMC4389590

[44] Hansen CG & Nichols BJ (2009) Molecular mechanisms of clathrin‐independent endocytosis. J.Cell Sci 122, 1713–1721. 19461071 10.1242/jcs.033951 PMC2723140

[45] Glebov OO , Bright NA & Nichols BJ (2006) Flotillin‐1 defines a clathrin‐independent endocytic pathway in mammalian cells. Nat Cell Biol 8, 46–54. 16341206 10.1038/ncb1342

[46] Park RJ , Shen H , Liu L , Liu X , Ferguson SM & De Camilli P (2013) Dynamin triple knockout cells reveal off target effects of commonly used dynamin inhibitors. J Cell Sci 126, 5305–5312. 24046449 10.1242/jcs.138578 PMC3828596

[47] Dutta D , Williamson CD , Cole NB & Donaldson JG (2012) Pitstop 2 is a potent inhibitor of clathrin‐independent endocytosis. PLoS One 7, e45799. 23029248 10.1371/journal.pone.0045799 PMC3448704

[48] Vercauteren D , Vandenbroucke RE , Jones AT , Rejman J , Demeester J , De Smedt SC , Sanders NN & Braeckmans K (2010) The use of inhibitors to study endocytic pathways of gene carriers: optimization and pitfalls. Mol Ther 18, 561–569. 20010917 10.1038/mt.2009.281 PMC2839427

[49] Pachetti M , Marini B , Benedetti F , Giudici F , Mauro E , Storici P , Masciovecchio C , Angeletti S , Ciccozzi M , Gallo RC *et al*. (2020) Emerging SARS‐CoV‐2 mutation hot spots include a novel RNA‐dependent‐RNA polymerase variant. J Transl Med 18, 179. 32321524 10.1186/s12967-020-02344-6 PMC7174922

[50] Chand GB & Azad GK (2020) Identification of novel mutations in RNA‐dependent RNA polymerases of SARS‐CoV‐2 and their implications. bioRxiv 2020, 10.1101/2020.05.05.079939 PMC7337032 32685291

[51] Mazzon M & Marsh M (2019) Targeting viral entry as a strategy for broad‐spectrum antivirals. F1000Res 8. 10.12688/f1000research.19694.1. PMC6743247 31559009

[52] Owens B (2020) Excitement around hydroxychloroquine for treating COVID‐19 causes challenges for rheumatology. Lancet Rheumatol 2, e257. 32368738 10.1016/S2665-9913(20)30089-8 PMC7193144

[53] Borba MGS , de Almeida Val F , Sampaio VS , Alexandre MA , Melo GC , Brito M , Mourao M , Sousa JD , Guerra MV , Hajjar L *et al*. (2020) Chloroquine diphosphate in two different dosages as adjunctive therapy of hospitalized patients with severe respiratory syndrome in the context of coronavirus (SARS‐CoV‐2) infection: preliminary safety results of a randomized, double‐blinded, phase IIb clinical trial (CloroCovid‐19 Study). medRxiv. 10.1101/2020.04.07.20056424.

[54] Letko M , Marzi A & Munster V (2020) Functional assessment of cell entry and receptor usage for SARS‐CoV‐2 and other lineage B betacoronaviruses. Nat Microbiol 5, 562–569. 32094589 10.1038/s41564-020-0688-y PMC7095430

[55] Otomo M , Takahashi K , Miyoshi H , Osada K , Nakashima H & Yamaguchi N (2008) Some selective serotonin reuptake inhibitors inhibit dynamin I Guanosine Triphosphatase (GTPase). Biol Pharm Bull 31, 1489–1495. 18670077 10.1248/bpb.31.1489

[56] Takahashi K , Miyoshi H , Otomo M , Osada K , Yamaguchi N & Nakashima H (2010) Suppression of dynamin GTPase activity by sertraline leads to inhibition of dynamin‐dependent endocytosis. Biochem Biophys Res Commun 391, 382–387. 19913505 10.1016/j.bbrc.2009.11.067

[57] Herxheimer H (1949) Antihistamines in bronchial asthma. Br Med J 2, 901–905. 15390552 10.1136/bmj.2.4633.901 PMC2051412

[58] Sharma RK , Sehgal S , Sachdeva N , Kumar R & Gupta A (2019) Direct engagement of TLR9 ligand with T helper cells leads to cell proliferation & up‐regulation of cytokines. Immunol Invest 48, 79–95. 30239236 10.1080/08820139.2018.1515223

[59] Mckendrick GD & Medlock JM (1958) Pulmonary moniliasis treated with nystatin aerosol. Lancet 271, 621–622. 10.1016/s0140-6736(58)90872-9 13515299

[60] Tomkiewicz RP , App EM , Zayas JG , Ramirez O , Church N , Boucher RC , Knowles MR & King M (1993) Amiloride inhalation therapy in cystic fibrosis: influence on ion content, hydration, and rheology of sputum. Am Rev Respir Dis 148, 1002–1007. 8214916 10.1164/ajrccm/148.4_Pt_1.1002

[61] Lin H‐P , Singla B , Ghoshal P , Faulkner JL , Cherian‐Shaw M , O'Connor PM , She JX , Belin de Chantemele EJ & Csányi G (2018) Identification of novel macropinocytosis inhibitors using a rational screen of Food and Drug Administration‐approved drugs. Br J Pharmacol 175, 3640–3655. 29953580 10.1111/bph.14429 PMC6109223

[62] Morgan EH & Iacopetta BJ (1987) Vinblastine but not other microtubule inhibitors block transferrin endocytosis and iron uptake by reticulocytes. Clin Exp Pharmacol Physiol 14, 119–126. 3608243 10.1111/j.1440-1681.1987.tb00965.x

[63] Inhaled Itraconazole Fast‐Tracked for Allergic Bronchopulmonary Aspergillosis in Asthma. Available at: https://www.pulmonologyadvisor.com/home/topics/asthma/inhaled‐itraconazole‐fast‐tracked‐for‐allergic‐bronchopulmonary‐aspergillosis‐in‐asthma/. (Accessed: 1st April 2020).

[64] Itraconazole Inhalation Receives IND Approval for Phase 2 Clinical Trial. Available at: https://www.pharmacytimes.com/resource‐centers/asthma/itraconazole‐inhalation‐receives‐ind‐approval‐for‐phase‐2‐clinical‐trial. (Accessed: 3rd April 2020).

[65] Irie T & Uekama K (1997) Pharmaceutical applications of cyclodextrins. III. Toxicological issues and safety evaluation. J Pharm Sci 86, 147–162. 9040088 10.1021/js960213f

